# A New Predictor Score for Postoperative Seizures in Brain Tumor Patients Without a Seizure History (BRAINNN Score)

**DOI:** 10.7759/cureus.84424

**Published:** 2025-05-19

**Authors:** Sergio Díaz-Bello, Marco Antonio Muñuzuri-Camacho, Luis Rodríguez-Hernández, Elvira Castro-Martinez, Eliezer Villanueva-Castro, Domingo Coutinho-Thomas, Rodolfo Villalobos-Díaz, German López-Valencia, Tomas Moncada-Habib, Ivan Abdiel Rodríguez-Hernández, Bernardo Cacho-Díaz, Guillermo Axayacalt Gutierrez-Aceves, Sergio Moreno-Jiménez, Alberto Gonzalez-Aguilar

**Affiliations:** 1 Neurosurgery, Instituto Nacional de Neurología y Neurocirugía Manuel Velasco Suárez, Mexico City, MEX; 2 Neurology, Instituto Nacional de Neurología y Neurocirugía Manuel Velasco Suárez, Mexico City, MEX; 3 Neurosurgery, Instituto Nacional de Neurología y Neurocirugía Manuel Velasco Suarez, Mexico City, MEX; 4 Medicine and Nutrition, Universidad de Guanajuato, Guanajuato, MEX; 5 Neurooncology, Instituto Nacional de Cancerología, Mexico City, MEX; 6 Radioneurosurgery, Instituto Nacional de Neurología y Neurocirugía Manuel Velasco Suárez, Mexico City, MEX; 7 Neurooncology, International Cancer Center, Mexico City, MEX

**Keywords:** anticonvulsants, brain neoplasms, predictive score, prophylaxis, seizures

## Abstract

Objective: To identify risk factors for postoperative seizures in patients with brain tumors without preoperative seizures and develop a predictive scoring system to guide antiepileptic drug (AED) prophylaxis.

Material and methods: A retrospective analysis was conducted on patients with intra-axial brain tumors and no history of preoperative seizures or AED use. Logistic regression identified significant predictors of postoperative seizures, and a scoring system was created using receiver operating characteristic (ROC) analysis. Data were drawn from a neuro-oncology database that had been active since 1970.

Results: A total of 446 patients were included from 16,918 records, with a mean age of 45.1 years (67.8% male, 345 with gliomas). Over 20% experienced postoperative seizures. Logistic regression identified five significant predictors, including tumor location, patient age, extent of resection, and neoplastic edema. A scoring system was developed to improve seizure risk assessment and seizure control. The BRAINNN Score (Brain tumor-associated Risk Assessment Index developed at the National Institute of Neurology and Neurosurgery, INNN) is a newly designed predictive tool to estimate the risk of postoperative seizures in brain tumor patients without a prior history of seizures.

Conclusions: This study proposes a personalized approach to AED prophylaxis using a novel, objective scoring system based on clinically relevant factors. This framework has the potential to optimize perioperative care and improve outcomes for patients with brain tumors.

## Introduction

Seizures are a common clinical manifestation of brain tumors, occurring in 15-50% of patients prior to diagnosis, with incidence influenced by tumor type, location, size, and patient age [1]. Despite their frequency, the prophylactic use of antiepileptic drugs (AEDs) in patients without a prior history of seizures remains controversial. Current recommendations discourage routine AED administration in seizure-naïve individuals and suggest tapering after the first postoperative week if no seizures occur [2].

This issue is further complicated by inconsistencies in international guidelines. The American Academy of Neurology (AAN), the Society for Neuro-Oncology (SNO), and the European Association of Neuro-Oncology (EANO) collectively advise against routine AED use in newly diagnosed, seizure-free brain tumor patients [3]. Similarly, the National Comprehensive Cancer Network (NCCN) discourages prophylactic AED use except in selected high-risk cases, leaving decisions largely to clinician judgment [4]. The Korean Society for Neuro-Oncology (KSNO) has adopted comparable recommendations, advising against prophylactic AEDs in the absence of seizures [5]. Notably, earlier AAN guidance also concluded that prophylactic AEDs do not improve outcomes in seizure-naïve patients [6]. This variability in expert opinion reflects a broader uncertainty in clinical practice and highlights the urgent need for objective tools to better identify patients genuinely at risk of postoperative seizures. Such tools would enable more tailored AED use and reduce unnecessary exposure to potential side effects.

Compounding the problem is the frequent lack of distinction in the literature between preoperative and postoperative seizure risk. Many studies explore tumor-related epilepsy in general terms, limiting their relevance to the postsurgical setting. However, the mechanisms driving postoperative seizures likely differ, encompassing surgical trauma, residual tumor, peritumoral edema, hemorrhage, or cortical irritation, and may not align with those that cause seizures preoperatively [7]. Tumor-specific characteristics such as histology and anatomical location are also believed to influence seizure susceptibility, though their precise roles remain insufficiently defined.

Based on these considerations, our central hypothesis is that specific clinical, radiological, and pathological features can objectively predict the occurrence of postoperative seizures in seizure-naïve patients undergoing brain tumor resection. Identifying these predictive factors would allow for the development of a practical risk stratification tool to guide individualized AED decision-making.

To date, no validated scoring system exists specifically for predicting postoperative seizures in this patient population. This lack of an evidence-based framework hinders consistent decision-making regarding AED prophylaxis. To address this gap, we conducted a retrospective, single-center study evaluating seizure-naïve patients who underwent brain tumor surgery. Our objectives were to identify clinical, radiological, and pathological variables associated with early postoperative seizures and to develop a predictive scoring model, the BRAINNN Score. The BRAINNN Score (Brain tumor-associated Risk Assessment Index developed at the National Institute of Neurology and Neurosurgery, INNN) is a newly designed predictive tool to estimate the risk of postoperative seizures in brain tumor patients without a prior history of seizures.

A tool of this nature holds substantial clinical potential. Facilitating risk-based AED prescribing, this tool may help reduce unnecessary drug exposure, minimize adverse effects, and improve individualized follow-up strategies within neuro-oncology practice.

## Materials and methods

Study design and patient selection

This retrospective, observational, single-center study analyzed data from the neuro-oncology database, which includes records of patients diagnosed with intracranial intra-axial brain tumors from 1970 to the present. The objective was to identify clinical, radiological, and pathological factors associated with postoperative seizures. The study specifically focused on seizure-naïve patients who underwent surgical resection, had no prior history of seizures or AED use, and had complete clinical and follow-up data available. Relevant demographic, clinical, and tumor-related variables, such as age, sex, tumor type, and location, were systematically collected to comprehensively characterize the cohort.

Significant peritumoral edema was defined as a midline shift greater than 5 mm and/or evidence of herniation on imaging, following the criteria described by Motuel et al. [8]. However, this threshold may vary between institutions. To improve reproducibility and objectivity, future iterations of the model will incorporate volumetric edema measurements (e.g., >64 cm³), obtained through automated MRI segmentation.

Molecular markers such as isocitrate dehydrogenase (IDH) mutation status, 1p/19q co-deletion, and O6-Methylguanine-DNA methyltransferase (MGMT) promoter methylation were not included in the current dataset. These biomarkers are increasingly recognized as important predictors of prognosis and seizure risk. Their inclusion in future iterations of the BRAINNN Score is warranted to align with contemporary neuro-oncological standards.

Statistical analysis: logistic regression model

A comprehensive multivariate logistic regression analysis was conducted to evaluate the association between a wide range of clinical and tumor-related factors and the likelihood of postoperative seizures. A total of 22 preoperative and intraoperative variables were considered for inclusion in the model, selected based on their clinical relevance and potential relationship with seizure risk. These variables included clinical factors such as age, sex, diabetes mellitus (DM), systemic arterial hypertension (SAH), corticosteroid use, Karnofsky Performance Status (KPS), preoperative motor deficits, cortical syndromes, and others. Tumor-related factors included tumor type, location, size, grade (according to the WHO classification), growth pattern (cortical or subcortical), presence of edema, hemorrhagic or calcified tumors, tumor resection type (partial or total), and the duration of disease progression.

The logistic regression model was constructed to isolate and quantify the independent effects of each factor, controlling for potential confounding variables. Odds ratios (ORs) with 95% confidence intervals (CIs) were calculated for each factor to assess the strength of its association with postoperative seizures. A significance level of p <0.05 was applied, and only factors with p-values below this threshold were considered significant.

Although a traditional logistic regression approach was used in the current analysis, we acknowledge that penalized regression methods, such as Least Absolute Shrinkage and Selection Operator (LASSO), could offer a more robust variable selection process by reducing multicollinearity and highlighting the most informative predictors. Future iterations of the model will consider incorporating LASSO to improve performance and interpretability.

Receiver operating characteristic (ROC) curve analysis

To further refine the model and develop a clinically relevant risk assessment tool, a ROC curve analysis was performed. This method evaluates the discriminatory power of a classification model by plotting the true positive rate (sensitivity) against the false positive rate (1 - specificity) across various threshold values of the predicted seizure risk scores. The Area Under the Curve (AUC) was calculated to quantify the model’s ability to differentiate between patients who did and did not develop postoperative seizures. An AUC greater than 0.8 was considered indicative of excellent performance.

The optimal cutoff point was determined by maximizing the Youden index, which identifies the threshold that best balances sensitivity and specificity. This cutoff was subsequently used to construct a risk scoring system aimed at supporting clinical decisions regarding postoperative seizure prevention and monitoring.

In addition to AUC analysis, future work will incorporate calibration curves (e.g., the Hosmer-Lemeshow test) and internal validation methods to assess the agreement between predicted probabilities and observed outcomes. The final model should also report sensitivity, specificity, and predictive values at clinically meaningful thresholds.

Hypothesis testing and statistical thresholds

To ensure the robustness of the findings, hypothesis testing was conducted for each factor included in the logistic regression model. A significance threshold of p <0.05 was applied to determine statistically significant results. Only factors with p-values below this threshold were included in the final model. Additionally, sensitivity analyses were performed by stratifying the data based on clinical variables such as tumor grade and age, ensuring the model remained stable and applicable across different patient subgroups.

Model validation and scoring system

After developing and validating the logistic regression model through ROC curve analysis, a risk scoring system was derived based on the most significant predictive variables. This system assigns a numerical score to each patient according to individual risk factors, stratifying them into low-, medium-, or high-risk categories. The scoring tool is designed to assist clinicians in guiding postoperative management and seizure surveillance. To mitigate potential overfitting and enhance the generalizability of the BRAINNN Score, future validation using an independent external cohort or stratified cross-validation is recommended. Internal validation through bootstrapping is also planned to evaluate model robustness and predictive performance.

## Results

**Figure 1:**
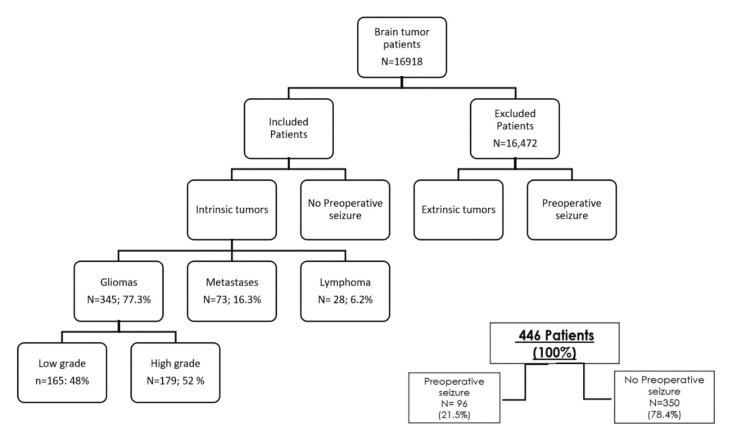
Profile of all patients with intracranial tumors Selection of patients with follow-up from 1979 to 2010 included in our retrospective analysis. From a total of 16,918 patients with a total final sample of 446 patients (96 in the group with preoperative seizures and 350 in the group without preoperative seizures), we excluded a total of 16,472 patients mostly for having preoperative seizures and extra-axial tumors and the others by because of missing data (absence of imaging studies, use of antiepileptics drugs chronically, incomplete demographics in a clinical file).

A total of 16,918 individuals were recorded in the Neuro-oncology database. The cohort for this study included 446 patients (Figure 1). The mean age was 45.1 years, 67.8% were male, and 345 patients had glioma (sociodemographic summary is given in Table 1).

**Table 1:** Data description of sociodemographic summary AED: Antiepileptic drugs.

Characteristic	Value	Percentage
Age (years)	48.1	
Sex	Female (144)	32.2
	Male (302)	67.8
Tumor Type	Glioma (345)	77.3
	Mets (73)	16.3
	Lymphoma (28)	6.2
Partial Resection	161	36
Complete Resection	285	64
Postoperative AED	Phenytoin (347)	77.8
	Carbamazepine (19)	4.3
	Primidone (9)	2
	Levetiracetam (71)	15.9
Number of Tumors	Alone (327)	73.4
	Multifold (119)	26.6
Location	Frontal (127)	28.4
	Temporal (87)	19.4
	Occipital (30)	6.8
	Parietal (40)	8.9
	Insular (42)	9.4
	Corpus callosum (46)	10.4
	Deep (74)	16.7
Seizures	Focal (210)	47.1
	F. Generalized Sec (155)	34.7
	Generalized (81)	18.2
Presentation Time (days)	3.9	
Significant Edema	139	31.2

**Figure 2:**
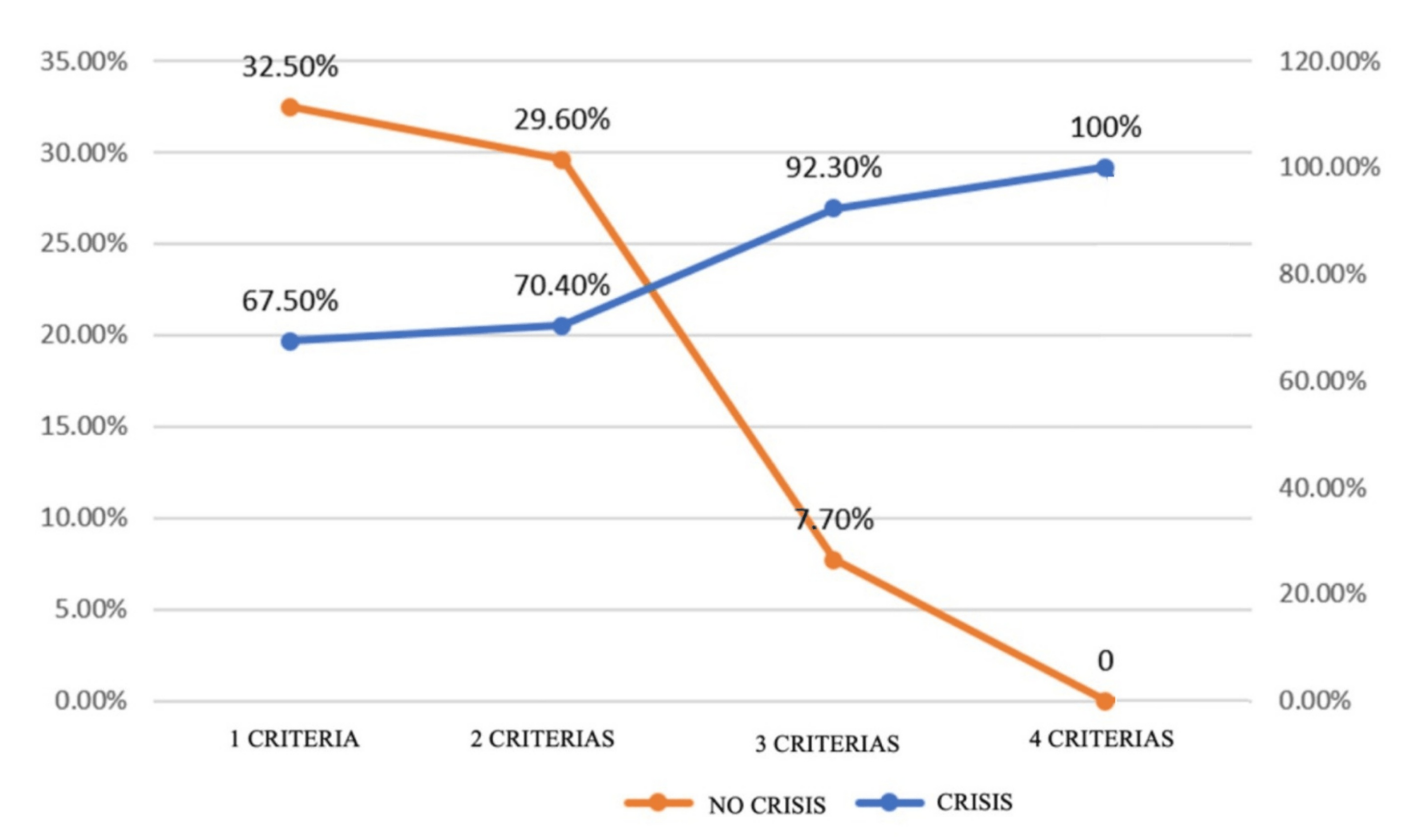
Correlation-number of criteria analysis The graph correlating the number of criteria present shows that the postoperative seizure presentation is directly proportional to the number of factors or predictors present.

Over 20% of patients presented with seizures after surgery. Through logistic regression, we evaluated 22 factors related with patients or tumors [age, significant edema, tumor type, localization, growth pattern, partial resection, hemorrhagic tumor, calcification tumor, sex, AEDs, KPS (Karnofsky performance status), only or multifold, DM (diabetes mellitus), SAH (systemic arterial hypertension), cortical syndromes, Depp syndrome, meningeal reinforcement, steroid use, evolution >three months, intracranial hypertension, motor deficits, grade (WHO). Only five factors were significant (Table 2 and Figure 2). Univariate analysis identified 11 variables with potential association to postoperative seizures (p < 0.1). However, multivariate logistic regression retained only five independent predictors: age <40 years, glioma histology, partial resection, significant edema, and frontal location. No statistically significant interactions were found between glioma histology and frontal location (p = 0.16). 

**Table 2:** Data description and assessment of the logistic regression analysis regarding significant risk factors for postoperative seizure occurrence CI: Confidence interval, OR: Odds ratio.

Variable	OR	95% CI	β (log OR)	SE	p-value	FDR-adjusted p
Age < 40 years	8.53	4.83 – 15.09	2.14	0.29	0.002	0.005
Glioma	9.38	3.78 – 23.30	2.24	0.39	0.0005	0.002
Partial resection	18.6	4.62 – 74.87	2.92	0.51	0.0032	0.006
Significant edema	3.23	1.15 – 9.09	1.17	0.47	0.0266	0.045
Frontal location	4.16	2.07 – 8.35	1.43	0.34	0.0001	0.001

**Figure 3:**
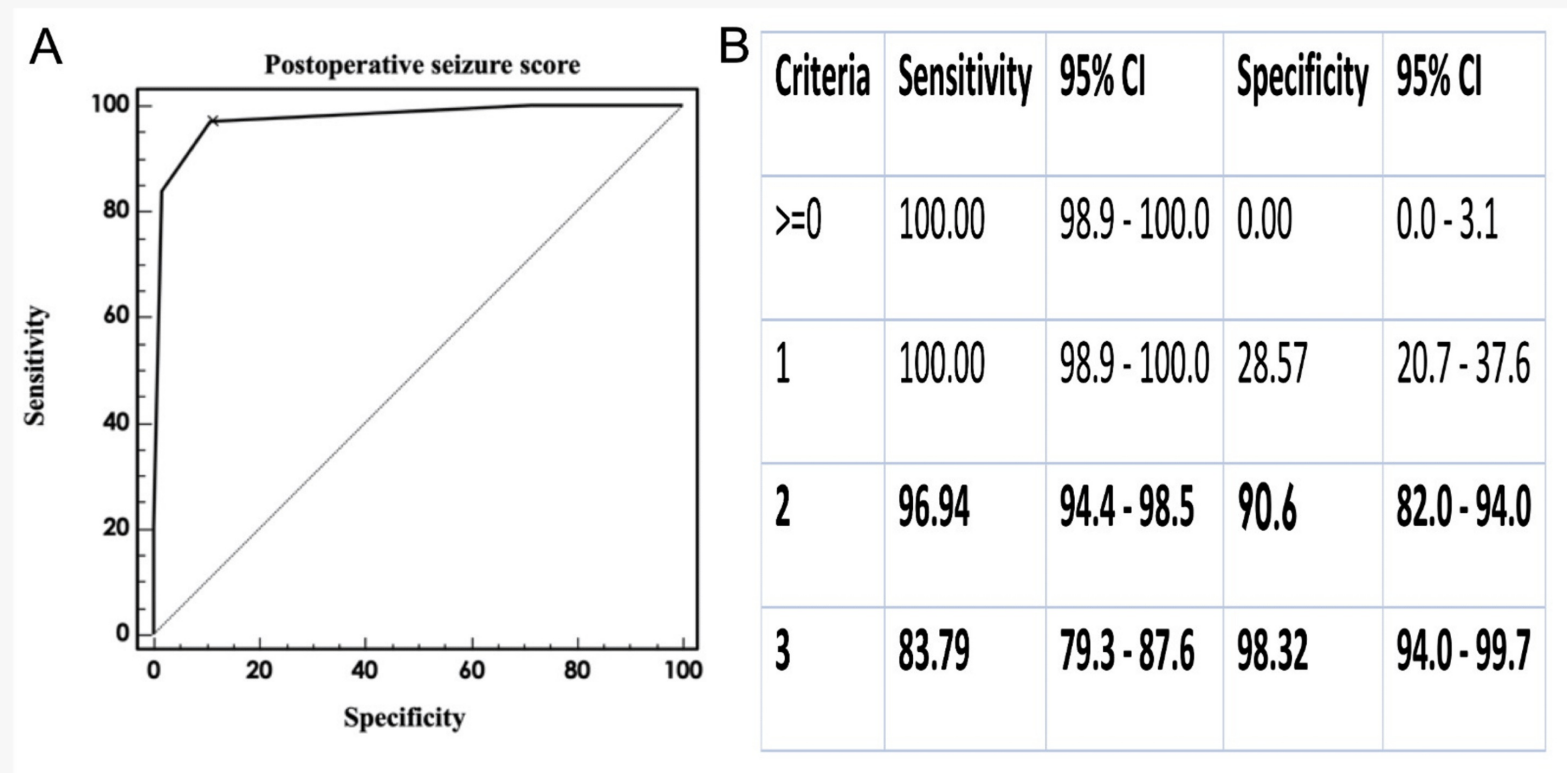
Receiver operating characteristic (ROC) curve The ROC curve illustrates excellent discriminatory performance of the BRAINNN Score, with an area under the curve (AUC) of 0.87 (95% CI: 0.83–0.91). At the optimal cutoff point, the model achieved a sensitivity of 84.2%, specificity of 79.1%, positive predictive value of 63.5%, and negative predictive value of 92.7%. These metrics indicate that the "Postoperative Score of CC" effectively identifies patients at risk of postoperative seizures, with a low rate of false positives and negatives. For reference, the diagonal line on the ROC plot represents random classification; curves above this line reflect better-than-random model performance.

Using the relevant factors, we calculated the ROC curve to develop the risk model scoring system for preoperative seizures (Figure 3). In addition to the ROC curve analysis, a calibration curve was generated to assess the agreement between predicted and observed seizure probabilities. The Hosmer-Lemeshow test yielded a p-value of 0.48, indicating good calibration.

**Figure 4:**
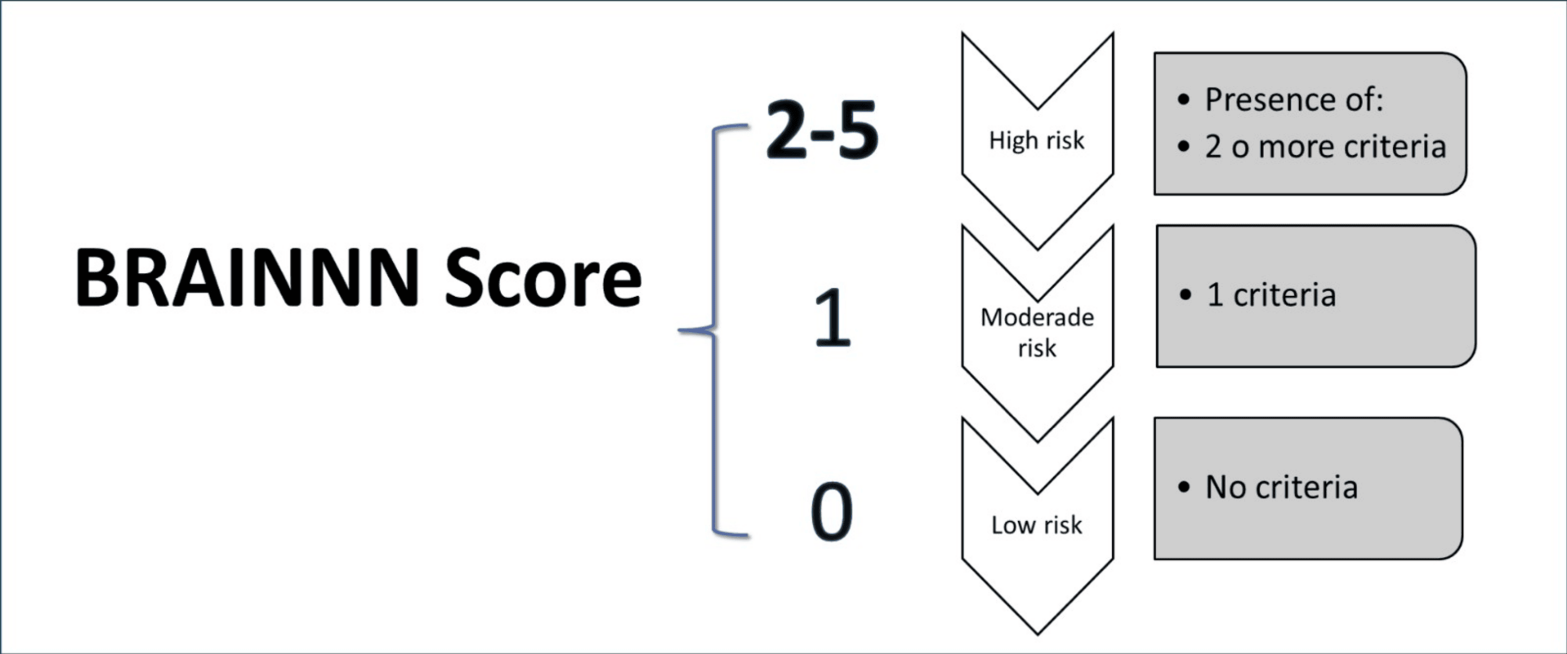
BRAINNN scale We consider that prophylactic treatment with AEDs should always be considered if the patient has high or moderate risk according to our scale and it is not justified at low risk.

Based on these results, we developed a scoring system to support therapeutic decision-making (Figure 4). 

Practical example of BRAINNN score application

A 35-year-old male patient presents with a frontal glioma, marked peritumoral edema, and has undergone a partial resection. He scores one point for each of the following risk factors: (a) Age <40 years, (b) Glioma histology, (c) Frontal lobe location, (d) Presence of significant peritumoral edema, (e) Partial tumor resection. His total score was 5 points. According to the BRAINNN Score, this places the patient in the high-risk category, supporting the clinical consideration of postoperative seizure prophylaxis with AEDs.

## Discussion

For many years, neurosurgeons have standardized antiepileptic prophylaxis in patients undergoing brain tumor resection, even though most evidence is neutral, neither in favor nor against prophylaxis of epileptic seizures in people with these pathologies [2]. These conclusions apply only to some AEDs and are ultimately guided by the assessment of individual risk factors for both patients and neoplasms under treatment.

In our study, the presence of postsurgical epileptic seizures in patients without preoperative seizures was 21.6%. These results are comparable with various studies, such as Al-Dorzi et al., showing a 14% incidence of crises after surgical treatment for brain tumor management [1]. Other studies have reported that post-craniotomy seizures during brain tumor management occur at different rates from 1.1 to 29%, possibly due to different combinations of cases (intra- and extra-axial tumors) of patients, which imposes mixed results since the biological and molecular behaviors in these two entities are different [2,7,9].

Significant predictors of postoperative seizures

In our study, several clinical and radiological factors were evaluated as potential predictors of postoperative epileptic seizures. Among these variables, the significant predictors identified were age under 40 years, glioma histology, partial resection, presence of significant peritumoral edema, and a frontal tumor location.

Studies such as Wirsching et al. evaluated other variables such as seizure history, previous radiation therapy, chemotherapy, and tumor size [10]. In contrast, our study did not evaluate tumor size as a predictor, which represents a limitation and may explain discrepancies with previous findings. The relationship between neoplastic size and seizure presence after surgical resection is unclear. Rapidly growing tumors, like size-independent gliomas, especially those in deeper structures, are most often reported to have seizures. Lee et al., found that a larger tumor size was associated with the presentation of seizures (odds ratio (OR), 1.31 per cm^3^; 95% CI, 1.06-1.60) in low-grade but not high-grade tumors [11], while Telfeian et al. [12], observed that a smaller glioblastoma tumor size was associated with increased risk of postoperative seizures, because more brain dissection is required to reach smaller tumors. This may explain greater post-resection seizures in these cases [12].

Tumor location and epileptogenicity

Other characteristics studied were tumor location. In our study, 28.4% (OR 4.15; 95% CI: 2.07-8.34; Q: 0.0001) of intrinsic tumors were located in the frontal lobe, and this location was the only one significantly associated with epileptic seizures. This contrasts with some studies, such as that by Oushy et al., where lobular involvement of the tumor showed no association with perioperative seizures. The lack of association between tumor location and the presence of perioperative seizures in Soliman's study may be attributable to the selection of the patient population (exclusion of some neoplastic locations) and the relatively small sample size [13].

Contrary to the above, the association between tumor location and seizure occurrence is well supported in the literature. Viet-Thang et al. found that patients with temporal lobe tumors were twice as likely to develop epileptic seizures; this ratio was only reflected in low-grade gliomas [14]. Telfeian et al. investigated postoperative seizures in patients with glioblastoma, concluding that resection of frontal tumors and smaller tumors seemed to increase the risk of postoperative seizures [12]. This is probably explained by the incidence of higher isocitrate dehydrogenase (IDH) in the frontal lobe (89%) as a reason for increased epileptogenicity in low-grade glioma in this region, observed by Stockhammer et al. [15].

Clinical implications of the BRAINNN Score

The rate of crisis in our postoperative glioma patients was 77%. However, we did not stratify in high or low grade as in the study of Skardelly et al. [16], where the highest seizure rate was observed in patients with low-grade gliomas prior to surgery (61%), followed by high-grade gliomas (29%), and metastasis (15%), consistent with previous studies showing IDH mutation was an independent predictor of epileptogenicity mainly in low grade gliomas [15-18]. Regarding the age variable, our study gave significant guidance for the development of postoperative seizures in young patients (<40 years) as are other studies such as Diego Garbossa et al., in which the only significant variable for the development of seizures was age; younger patients had increased risk of seizures in the six months after surgery [19]. This is associated with previous studies showing the threshold for epileptic seizures increases with age [18-20]. It has also been shown that young people have a much lower convulsive threshold than elderly individuals. In addition, younger patients are more likely to develop a higher number of symptoms and triggers than older patients [21]. In children and adolescents, brain tumors, which are predominantly low-grade, have been shown to be more epileptogenic than high-grade ones that predominate among adults [21].

The degree of resection in our study showed significant findings (OR: 18.60; 95% CI: 4.62-74.87; Q: 0.0032), similar to those previously reported in the literature in which patients with subtotal resection or biopsy alone were more likely to suffer perioperative seizures in comparison to patients with gross total resection [13,16]. Significance was also found with respect to neoplastic edema (OR: 3.22; 95% CI: 1.460-9.08; Q: 0.0266). Unlike the criterion used by Skardelly et al., in which the total volume (>64 cm^3^) measured by radiological studies was used as a predictive factor of postoperative epileptic seizures [16], we took into account the edema's potential to displace and compress adjacent parenchymal structures. This decision was based on the multiple mechanisms of epileptogenicity in brain tumors, caused by direct effects of the tumor (tumorometric) or due to changes in the extracellular environment that cause cortical hyperexcitability (epileptocentric) [1,2,7].

Biological mechanisms and pathophysiology

The direct mass effect of the tumor alters the surrounding brain through edema, vascular insufficiency, and inflammation. The peritumoral cortex in patients with epileptic seizures reveals changes in synaptic vesicles and glial space junctions. An increase in CX43 protein expression has been found in perilesional tissue of seizures associated with brain tumors, suggesting an increase in astrocytic junctions [22,23]. There is persistence of neurons in the white substance, which is sometimes related to tumor pathology, and may predispose these patients to develop crises [23]. Peritumoral pH is significantly elevated compared to the normal cortex. This is believed to increase the likelihood of seizures by multiple mechanisms, including blocking internal currents of K+, inhibiting gamma-aminobutyric acid (GABA) conductance, and abolishing inhibition of NMDA receptors [24,25]. Studies on the micro-organization of the peritumoral cortex have revealed the loss of inhibitory synapses in pyramidal neurons.

Limitations and future directions

The effectiveness of seizure prophylaxis in controlling postoperative seizures after craniotomy for tumor resection remains unclear. Most patients do not have seizures before surgery, and to prevent seizures, it is common to treat patients operated on for brain tumors post-surgically with different AEDs without a statistically significant objective parameter. A 2020 Cochrane review examined AED prophylaxis after craniotomy, regardless of surgical indication [26]. Unfortunately, current evidence was limited by different methodologies, heterogeneous pathologies, and inconsistent reports. The prophylaxis after craniotomy, specifically for brain tumors, is not supported [27,28]. However, these trials used older AEDs and often examined the post-diagnosis period, rather than strictly after surgical resection. In addition, a large number of these studies lack a statistical plan with power analysis to determine the appropriate sample size.

Given the controversy in this area, the prophylactic use of AEDs is still quite widespread. An online survey by the American Association of Neurological Surgeons found that while 63% of respondents 'almost always' prescribe postoperative AED for a supratentorial tumor, only 38% believe that treatment significantly reduces the risk of postoperative seizures [29]. In our local survey, 93% of neurosurgeons and 70% of neurologists would leave prophylaxis just by analyzing a tomographic study showing significant size and peritumoral edema displacing midline structures.

One major limitation of our study is the absence of systematic electroencephalography (EEG) monitoring, which may have resulted in an underestimation of non-convulsive seizures. This limitation, also noted by Elf et al., who used continuous EEG monitoring in 100 consecutive patients with primary brain tumors, could affect the external validity of our findings [30]. Additionally, as a retrospective study, there are inherent limitations related to potential selection biases and the inability to control for all confounding factors. The lack of prospective data also limits the generalizability of our findings to other populations and clinical settings.

Practical implications

Most published studies on post-surgical crisis prophylaxis included heterogeneous populations: intra- and extra-axial brain tumors, aneurysms, or trauma. Skardelly et al. demonstrated that the neoplastic volume/edema and the histological degree should be considered as predictive factors of crisis [16]. However, only a prognostic scale is proposed and not a reference or therapeutic scale that guides in decision-making for drug use, such as that presented by our group [17].

Unlike previously published work, our goal was not to determine whether AED prophylaxis is appropriate in postoperative intra-axial tumor patients. Instead, we emphasize the need for individualized decisions based on a structured, objective tool. The BRAINNN Score is the first tool proposed as a clinical decision-making guide, and we propose its future external validation, potential digital adaptation (e.g., web calculator), and integration into clinical workflows.

## Conclusions

Postoperative seizures remain a significant clinical challenge in patients undergoing resection of intra-axial brain tumors. This study introduces the BRAINNN Score, providing a preliminary objective basis for stratifying postoperative seizure risk based on clinical and radiological factors such as tumor location, extent of resection, and peritumoral edema. Our findings underscore the importance of an individualized, evidence-based approach to managing seizure risk, enabling clinicians to make more tailored decisions regarding prophylactic AED use, potentially reducing unnecessary treatments and improving patient outcomes.

Importantly, the BRAINNN Score represents the first quantifiable predictive tool specifically designed for patients with intra-axial tumors who have no prior history of epilepsy, marking a novel contribution to the field. However, it is essential to recognize that the score requires external validation before clinical implementation. Future research should focus on multicenter validation, the incorporation of molecular biomarkers, the systematic use of postoperative EEG, and the development of a digital version of the score to enhance its clinical utility and facilitate broader adoption.
